# Treatment and diagnostic challenges associated with the novel and rapidly emerging antifungal-resistant dermatophyte, *Trichophyton indotineae*

**DOI:** 10.1128/jcm.01407-24

**Published:** 2025-06-11

**Authors:** Yasmeen Vincent Marbaniang, Daniela Leto, Huda Almohri, Mohammad Rubayet Hasan

**Affiliations:** 1 Medical and Scientific Department, LifeLabs 149910 , Toronto, Canada; 2 Department of Pathology and Molecular Medicine, McMaster University 3710, Hamilton, Canada; 3 Hamilton Regional Laboratory Medicine Program 709550, Hamilton, Canada; 4 Research Institute at St. Joe’s Hamilton, Hamilton, Canada; Vanderbilt University Medical Center, Nashville, Tennessee, USA

**Keywords:** dermatophyte, *Trichophyton indotineae*, antifungal resistance, diagnosis, antifungal therapy

## Abstract

*Trichophyton indotineae* is a recently discovered dermatophyte species that causes recalcitrant dermatophytosis. It was first reported from India and has quickly spread across the globe. The exact prevalence of *T. indotineae* remains unknown due to limited surveillance. It has reached epidemic proportions in the Indian subcontinent. In India, this new species has largely replaced other previously common dermatophytes. Reports from Western countries suggest most cases are imported, with some reports of local transmission. A recent report from the United Kingdom indicates that *T. indotineae* now accounts for 38% of dermatophyte isolates tested in their national referral laboratory. *T. indotineae* causes widespread, inflammatory dermatophytosis affecting large areas of the body. Dermatophytosis caused by *T. indotineae* is difficult to manage due to the limited availability of mycology laboratories capable of reliably identifying and performing antifungal susceptibility testing, and because of its resistance to commonly used antifungals. Culture and physiological characteristics cannot confirm identification to the species level, requiring species-level confirmation by molecular methods like internal transcribed spacer sequencing. It is important for clinicians and mycology laboratories to be aware of and consider the possibility of *T. indotineae* infection in patients with relevant demographic, clinical, and travel history. This would decrease delay in diagnosis, prevent inappropriate use of medications like steroids and ineffective antifungal agents, and provide opportunities to make recommendations for good hygiene practices to prevent transmission. In this mini review, we describe the emergence of *T. indotineae*, its diagnostic and treatment challenges, and the current state, and provide recommendations for future direction.

## INTRODUCTION

Dermatophytosis is a superficial mycosis of the skin, hair, and nails caused by dermatophytes, which are keratinophilic fungi (1). *Trichophyton* species causes the majority of dermatophyte infections (2, 3). Infections are acquired through contact with infected humans, animals, soil, or contaminated fomites (4). Dermatophyte infections have generally responded well to first-line oral antifungal agents such as terbinafine (TRB), griseofulvin, and azoles (5). However, since 2016, Indian dermatologists started reporting widespread, chronic dermatophytosis resistant to first-line antifungal treatment, notably TRB (6–8). These isolates were negative for both urease and hair perforation tests (HPTs). Subsequently, these isolates were identified as *Trichophyton mentagrophytes* genotype VIII (2, 9). In 2020, based on clinical, mycological, and molecular features, this TRB-resistant *Trichophyton* species belonging to *T. mentagrophytes* genotype VIII was described as a novel species and designated as *Trichophyton indotineae* (10) to indicate the country (India) from where it was first reported. However, in a recent publication, the appropriateness of the name *T. indotineae* has been questioned, with two major concerns: the pejorative connotation of the term indotineae and inadequate information regarding the origin of this novel dermatophyte (11).

Over the past decade, India has experienced an alarming rise in *T. indotineae* infections.

A recent survey revealed that 78% of dermatophyte cases in the country are caused by *T. indotineae* (12). It is hypothesized that widespread availability and misuse of topical corticosteroid creams and antifungal agents in India contributed to the emergence of this infection (1). *T. indotineae* causes extensive, recalcitrant tinea cruris and tinea corporis, including atypical locations such as genitalia (12). Now endemic to both India and Iran, *T. indotineae* has also been identified in imported cases worldwide, with occasional reports of limited local transmission, highlighting the risk of epidemic spread to more countries in the near future (13, 14). The rapid emergence and importation of this drug-resistant dermatophyte to many countries present a significant public health problem, highlighting the urgent need for better diagnostic tools and methods for the detection of *T. indotineae* and better treatment options.

In this mini review, we summarize the existing literature on *T. indotineae*, with a particular focus on diagnostic and treatment challenges, currently available diagnostic and treatment modalities, and future directions. Our aim is to improve awareness and promote better diagnostic practices to support prompt and appropriate treatment, implement effective public health measures to prevent further spread, and decrease healthcare costs due to this infection.

## THE EMERGENCE OF *T. INDOTINEAE*

The reported increase in the incidence of TRB-resistant *Trichophyton* spp. causing chronic, recalcitrant dermatophytosis in India was initially attributed to *Trichophyton interdigitale*, an anthropophilic offshoot of the zoophilic *T. mentagrophytes*. High levels of resistance to TRB in these isolates were linked to point mutations in the squalene epoxidase gene (*SQLE*) (15).

A unique clade related to the *T. mentagrophytes/interdigitale* complex was confirmed by genome analysis (16). Phylogenetic studies identified unique single-nucleotide polymorphisms (SNPs) in the ribosomal internal transcribed spacer (ITS) region of these isolates and designated them “*Trichophyton mentagrophytes* ITS Type VIII” (9, 17). However, these isolates were negative for both the urease and HPT, which are uncharacteristic of *T. interdigitale* and zoophilic *T. mentagrophytes* (1). This ultimately led to the formal designation of a new species, *T. indotineae* (10).

It is speculated that the misuse of topical glucocorticoids and antimycotic/antibiotic fixed-dose combination creams, frequently containing potent steroids like clobetasol propionate, has led to the adaptation of *T. indotineae* infections and therapy recalcitrant dermatophytosis. These creams are widely accessible over the counter in India and other countries, such as in African countries and the Middle East. They are affordable and often recommended by pharmacists (3, 18–20). Prolonged steroid use often results in steroid-modified tinea lesions, such as tinea pseudoimbricata (*21*), leading to misdiagnosis and delay in treatment. Additionally, steroid misuse can cause systemic effects like Cushing’s syndrome (22) and contribute to recurrent and chronic dermatophytosis by potentially disrupting the skin microbiome and immune response (23).

**Fig 1:**
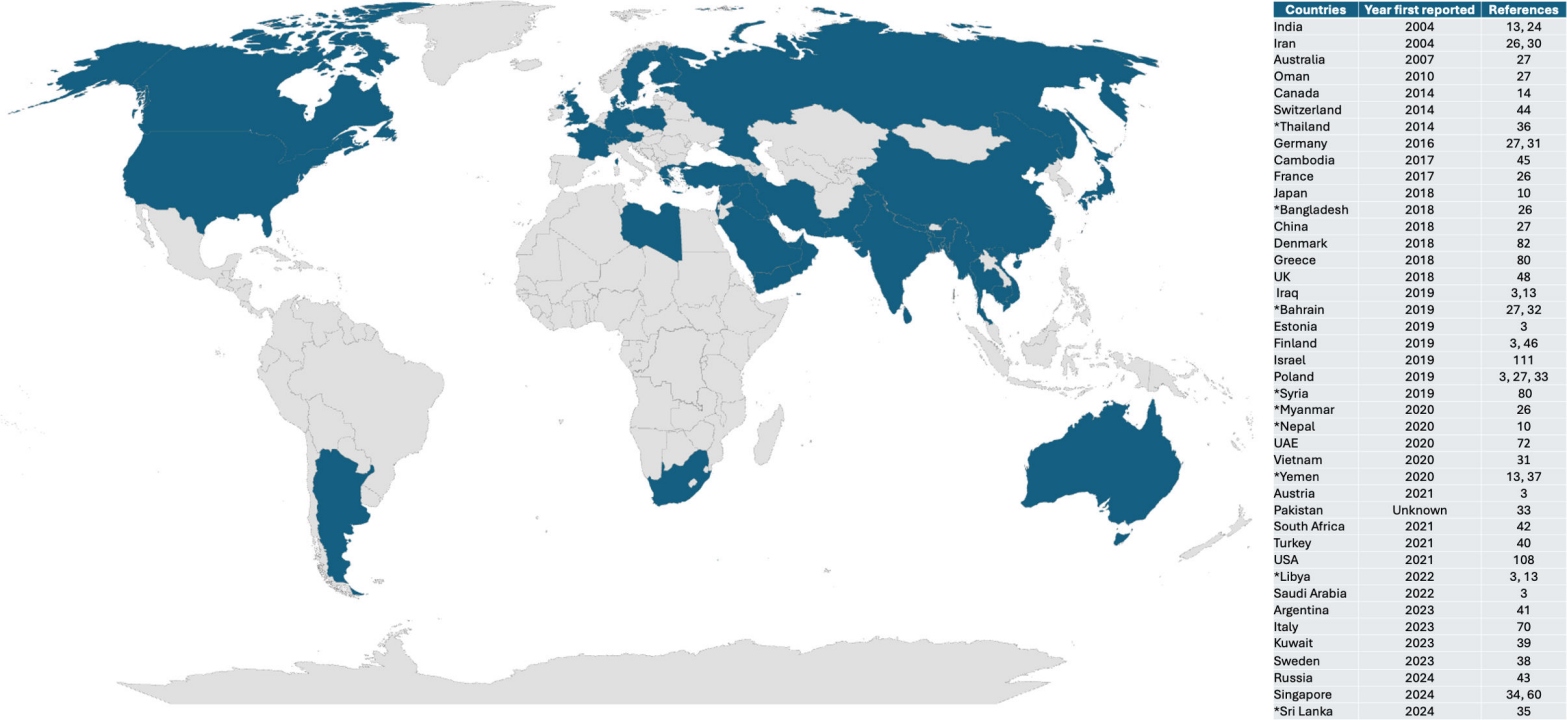
Worldwide distribution of *T. indotineae*. Shaded areas represent countries where *T. indotineae* has been identified in patients with dermatophytosis or where the infection was most likely acquired. The years shown for each country in the table represent the earliest known year when *T. indotineae* was isolated in that country. *Countries where the infection was most likely acquired but reported from a different country. The map was generated in Microsoft Excel (31–45)

*T. indotineae* has been present for more than a decade before it was first reported from India, as evidenced by the fact that a sequence corresponding to *T. indotineae* was identified in a GenBank sequence deposited in 2004 from India. This isolate was from a human skin sample (13, 24, 25). *T. indotineae* sequences were also deposited in GenBank from other countries before the first report of chronic recalcitrant dermatophytosis in 2016. In 2008, sequences of *T. indotineae* were deposited as *Arthroderma benhamiae* from Japan and Australia (26). By 2017–2019, *T. indotineae* had replaced the previously dominant dermatophytes across India. However, there was variability in the TRB resistance rate of *T. indotineae* between different regions of India. The national resistance rate overall was 72%–75%; however, in the Southern region, it was much lower at 16%. Isolates from the Western region of India had a resistance rate of 77%, those from the Northern region had a resistance rate of 76%, and in the Eastern region, the resistance rate to TRB was 75% (12). Beyond the Indian subcontinent, *T. indotineae* has been reported from many countries (3, 13, 24, 27, 28) (Fig. 1). It has become endemic in Iran (29) with ITS sequences confirming its presence as early as 2004 (26, 30). A multicenter study in Iran between 2016 and 2018 showed TRB resistance in 18% of *T. indotineae* isolates, with some isolates having minimum inhibitory concentrations (MICs) as high as 32 µg/mL (13, 29). Similar trends have been observed in Iraq, where *T. indotineae* accounted for 69.2% of isolates in a 2019 survey (13).

*T. indotineae* spread is primarily associated with migrants from and travelers to the Indian subcontinent, including Pakistan, Bangladesh, Sri Lanka, Nepal, and Myanmar (3, 26, 27, 46, 47). ITS sequencing has confirmed its endemicity across a broad geographic region, suggesting an endemic area extending from the Middle East to Southeast Asia (13). Most recently, studies conducted in Canada and the United Kingdom have reported a sharp increase in *T. indotineae* cases over the past few years (14, 48). In the United Kingdom, this organism now accounts for 38% of dermatophyte isolates referred to the National Mycology Reference Laboratory (48). Despite the growing number of reports, robust epidemiological studies assessing incidence and prevalence over time remain limited (14, 48).

## GENOTYPIC AND PHENOTYPIC CHARACTERISTICS OF *T. INDOTINEAE*

Dermatophytes are broadly categorized into three groups based on their habitat: anthropophiles (human-associated), zoophiles (animal-associated), and geophiles (soil-associated) (49–51). However, these distinctions can overlap, as some species adapt to different hosts and change their preferred habitat (52–54). The nomenclature of dermatophytes was revised in 2017, with an emphasis on genetic markers in addition to other parameters such as microscopic features, culture and physiological characteristics, ecological niche, and propagation form (51). The ITS region of the rDNA is currently used to decisively identify dermatophytes to the species level based on SNPs (17, 55).

The taxonomic classification of *T. indotineae* has been challenging due to its morphological similarities in laboratory cultures with *T. interdigitale* and *T. mentagrophytes*. Between 2008 and 2016, these species were not clearly distinguished, with classification primarily based on their anthropophilic or zoophilic origin. *T. interdigitale* was considered an anthropophilic species, while *T. mentagrophytes* was regarded as zoophilic (9, 17). However, a 2017 revision reinstated species distinctions, and a 2019 study identified multiple genotypes associated with infection sources, geographic origins, and clinical presentations (17). Currently, six *T. interdigitale* and 22 *T. mentagrophytes* genotypes have been described, differing by up to nine nucleotides in their ITS sequences. Based on clinical criteria, Indian strains identified as “genotype VIII” via rDNA ITS likely belong to *T. interdigitale* due to probable human-to-human transmission, though their relatively high virulence suggested an animal origin. This taxonomic debate was resolved in 2020 when Kano et al. (10) described TRB-resistant cases in Indian and Nepali patients and proposed the name *T. indotineae*, based on ITS region sequences and other clinical and mycological features. Molecular studies, including multi-locus analyses by Tang et al., confirmed *T. indotineae* as a distinct species, genetically similar to *T. interdigitale* and *T. mentagrophytes*, and classified it as an anthropophilic offshoot within the T*. mentagrophytes* complex (TMC) (1, 3, 56, 57).

Whole-genome sequencing (WGS) of *T. indotineae* has been conducted in many countries, including India, Japan, Singapore, and the United States, albeit with limited studies and isolate numbers (58–61). These studies highlight the genetic basis of its drug resistance and pathogenicity. In India, genome sequencing of two clinical isolates achieved high coverage and identified a c.1342G>A mutation, causing an A448T substitution in *erg*1, which encodes SQLE, potentially linked to TRB and azole resistance (59, 62). Comparative genomic analysis revealed conserved architecture with *T. interdigitale* and *T. mentagrophytes*, highlighting genes involved in virulence and pathogenicity, including lipases, proteases, and cytochrome P450 enzymes. Single amino acid polymorphisms in key virulence proteins, such as Sub3 and Sub6, were suggested as potential phylogenetic markers (59). In Japan, multiple studies explored azole resistance mechanisms. WGS identified two types of azole-resistant strains with tandem repeats of *TinCYP51B*: type I with 2,404-bp repeats and type II with 7,374-bp repeats. Using CRISPR/Cas9, reducing the *TinCYP51B* copy number restored azole susceptibility, confirming that overexpression of *TinCYP51B* mediates resistance (61). Another study found overexpression of *TinMDR3* and *TinCYP51B* in resistant strains, with *TinCYP51B* amplification playing a predominant role in azole resistance. In Singapore, WGS of two isolates revealed a high genetic similarity with other South Asian isolates, with an SNP distance of 92 between them. The isolates lacked mutations in SQLE and *CYP51* gene families, correlating with phenotypic drug susceptibility. The study emphasized the underreported global prevalence of *T. indotineae* (60). Most recently, WGS of 11 *T. indotineae* isolates in the United States revealed that they were genetically distinct from *T. indotineae* isolates from India. Based on the travel history of the patients, WGS traced the origin of these isolates to Bangladesh, suggesting that *T. indotineae* may be endemic there (58). Additionally, SNV data indicated possible household transmission or acquisition from a shared source in two patients, while other isolates exhibited a variable number of SNVs, suggesting independent introductions of *T. indotineae* or its variants within New York City (58). Collectively, these studies underscore the genetic diversity of *T. indotineae*, its emerging global prevalence, and the complex mechanisms driving antifungal resistance, with WGS serving as a vital tool for monitoring and managing this pathogen (58–61).

**Fig 2:**
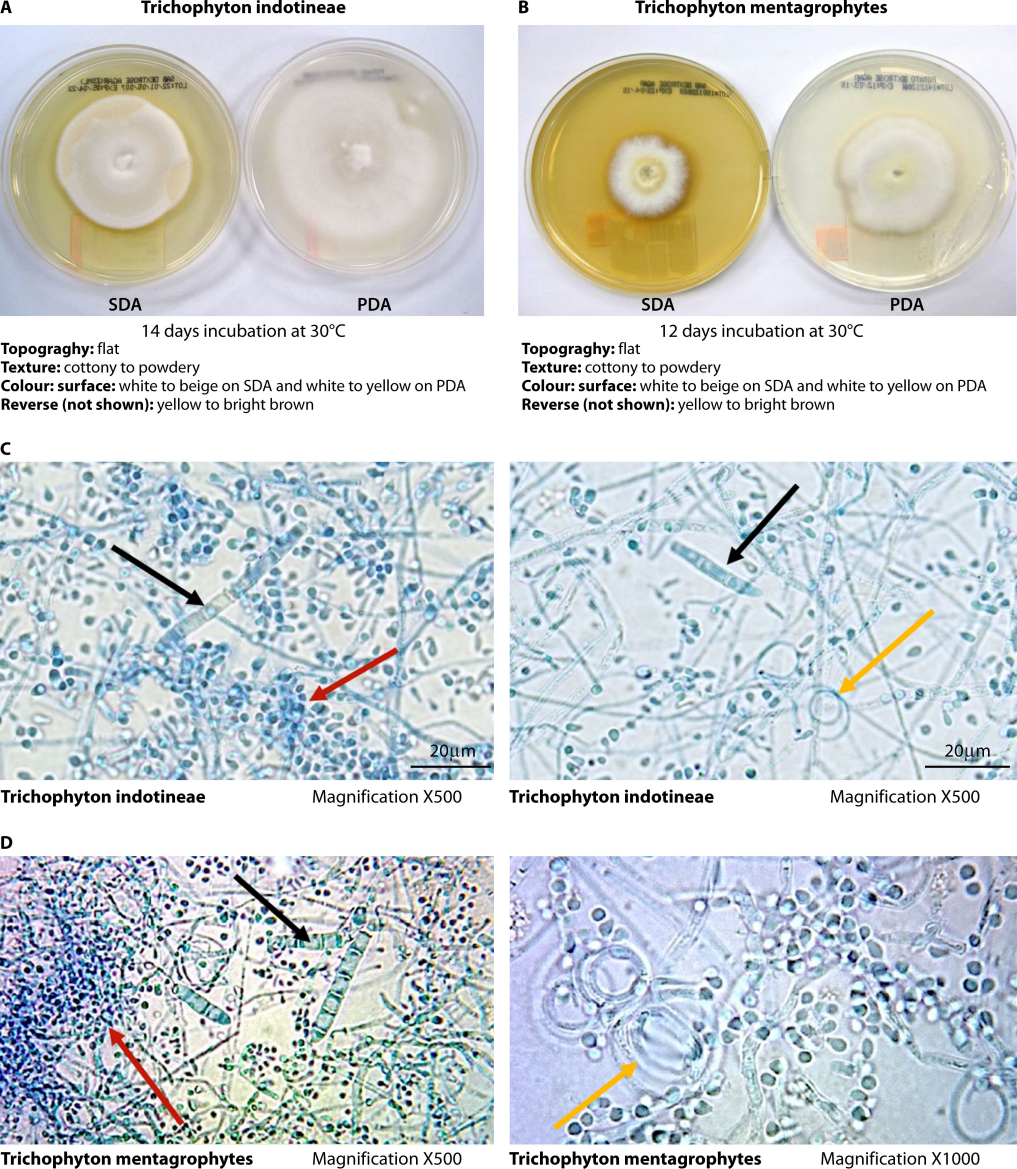
*T. indotineae* microscopy and culture. (**A**) *T. indotineae* colonies on Sabouraud dextrose agar (SDA) and potato dextrose agar (PDA) plates after 14 days of incubation at 30°C; (**B**) *T. mentagrophytes* colonies on Sabouraud dextrose agar (SDA) and potato dextrose agar (PDA) plates, after 12 days of incubation at 30°C; (**C**) Scotch tape preparation of *T. indotineae* stained with lactophenol cotton blue; (**D**) Scotch-tape preparation of *T. mentagrophytes* stained with lactophenol cotton blue; red arrow, small and big round microconidia in grape-like clusters; black arrow, cigar-shaped to club-shaped macroconidia; and orange arrow, spiral hyphae. Culture and identification tests were performed at Lifelabs Ontario Microbiology Laboratory, Toronto, ON, Canada

Both *T. indotineae* and *T. mentagrophytes* produce colonies that are flat, white to beige, cottony to powdery colonies with reverse yellow to bright brown on Sabouraud dextrose agar and potato dextrose agar (PDA) culture medium after 12–14 days of incubation at 30°C (3, 63). These macroscopic features are not species-specific (Fig. 2A and B) (63).

Microscopic examination of the colonies of both *T. indotineae* and *T. mentagrophytes* demonstrates the presence of small and big round microconidia in grape-like clusters, few subspherical to pyriform to clavate microconidia together with cigar-shaped to club-shaped macroconidia with three to six septa, smooth and thin-walled measuring 6–8 × 20–50 µm with narrow attachment bases. Spiral hyphae can be formed in older cultures (Fig. 2C and D) (3, 63).

**Fig 3:**
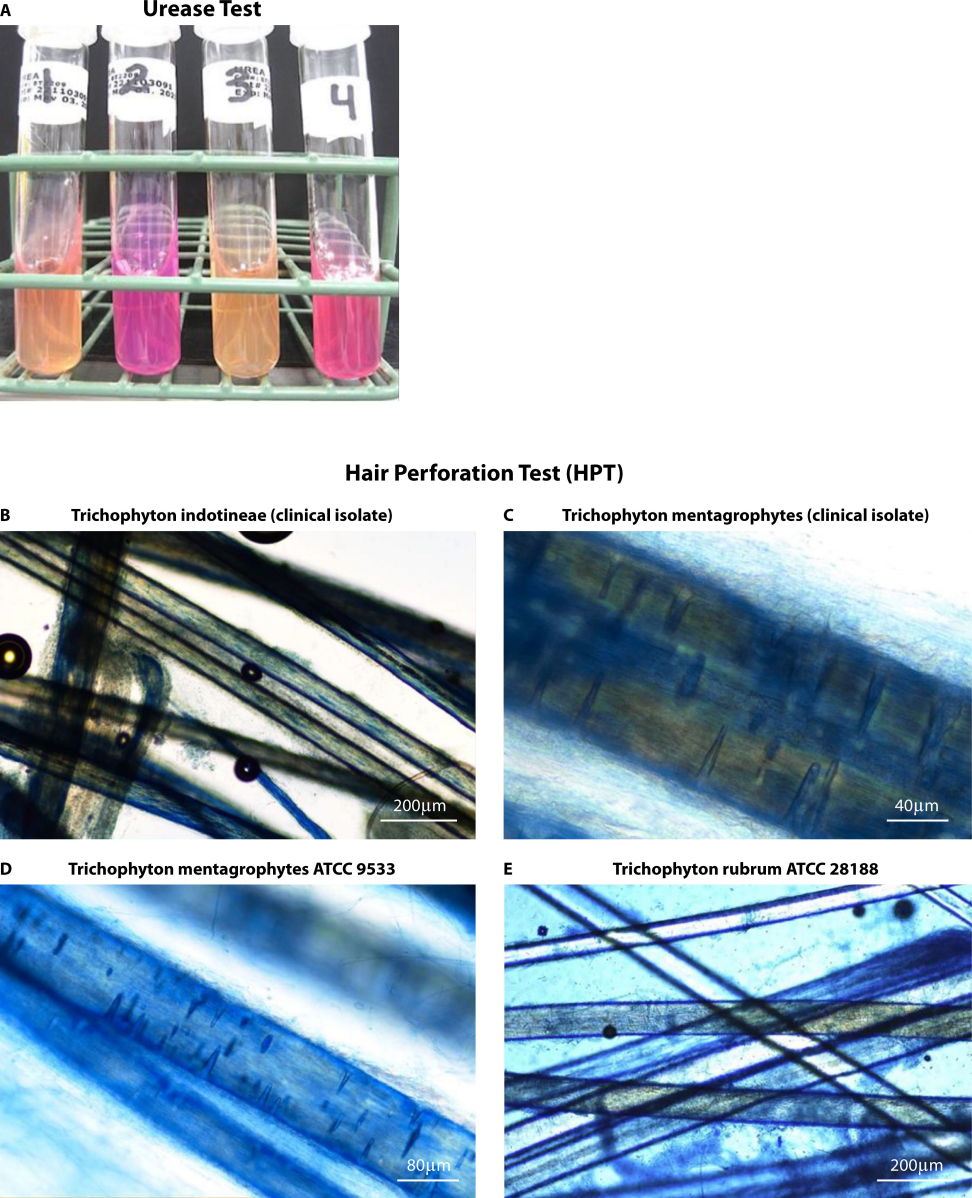
Physiological tests used for the identification of *T. indotineae*. (**A**) Urease test after 7 days of incubation at 30°C. Christensen urea agar urease positive result indicated by red-pink color and urease-negative result indicated by no color change. Tube 1: *T. indotineae* (clinical isolate); Tube 2: *T. mentagrophytes* (clinical isolate); Tube 3: *T. rubrum* ATCC 28188, negative quality control (QC); Tube 4: *T. mentagrophytes* ATCC 9533, positive QC. (**B**) *T. indotineae* (clinical isolate) HPT-negative; (**C**) *T. mentagrophytes* (clinical isolate) HPT-positive; (**D**) *T. mentagrophytes* ATCC 9533, HPT-positive QC; (**E**) *T. rubrum* ATCC 28188, HPT-negative QC. Culture and identification tests were performed at Lifelabs Ontario Microbiology Laboratory, Toronto, ON, Canada

*T. interdigitale* and *T. mentagrophytes* share physiological similarities that distinguish them from *T. indotineae* (1). *T. indotineae* is usually urease-negative or weakly positive and negative in the HPT, which differentiates it from *T. interdigitale* and *T. mentagrophytes*, both of which are usually urease-positive and HPT-positive (1, 63). (Fig. 3A through E).

Lipolytic activity varies significantly among dermatophyte species (1). For instance, *T. mentagrophytes* exhibits lipolysis, whereas *Trichophyton rubrum* does not. The lipolytic abilities of *T. mentagrophytes* and *T. interdigitale* were similar and higher than those of *T. indotineae*. This difference might correlate with the higher prevalence of *T. mentagrophytes* on lipid-rich areas such as the human scalp. Previous studies have linked hair perforation ability with lipolytic capacity in *T. mentagrophytes* and *T. interdigitale*. Both species showed significantly greater hair perforation ability compared to *T. indotineae*. Additionally, keratin degradation was notably higher in *T. interdigitale* than in *T. mentagrophytes* and *T. indotineae* (1)*.*

## TRANSMISSION AND CLINICAL CHARACTERISTICS

Current evidence suggests that *T. indotineae* is primarily transmitted from person to person. Family-based transmission has been documented in Iran, where an affected family with no travel history to India experienced infection (3). The species has also been identified in various provinces across Iran. In Germany, intra-familial transmission was observed in a couple originally from Iraq and in multiple cases involving international connections (46). These included a baby from Bahrain with tinea corporis who transmitted the infection to several family members, a German woman and her husband from Saudi Arabia, and a Libyan man who infected his partner and their child (3). Recently, sexual transmission was described in an immunocompetent woman in Pennsylvania, USA (64). Animals could also serve as reservoirs of infection based on sequence analysis of two sequences from calves that came from Egypt, one sequence from a dog from India, and three sequences from Poland with an unidentified animal host (26). Although there are no reports of zoonotic transmission yet, these results suggest that animals can serve as reservoirs of infection, and the possibility of zoonotic transmission should be considered. The limited occurrence in animals has led to the hypothesis that *T. indotineae* has undergone “anthropozation,” shifting from an animal-associated pathogen to one primarily adapted to human hosts (65). Indirect transmission is also possible via contaminated surfaces, pools, spas, locker rooms, shared living spaces, fomites such as towels, bedding, nail appliances, and hairbrushes (3, 66).

*T. indotineae* dermatophytosis can be severe, covering large areas of the body, and challenging to treat. They can present as extensive, pruritic plaques, minimally inflammatory, primarily affecting the trunk, extremities, and groin, causing tinea corporis, tinea cruris, tinea faciei, and tinea genitalis. Central clearing is often absent, and plaques may resemble eczema, and post-cure pruritus has frequently been reported. Dark-skinned individuals can present with prominent pigmentation (67). Lesions may present in various atypical morphologies, including erythematous, scaly concentric plaques with a “pseudoimbricata” appearance, papulosquamous, pustular, or steroid-modified tinea forms (65). Patients often report a history of ineffective treatment with topical or oral antifungals, including TRB, and some may have recently traveled to or been in contact with individuals from countries where *T. indotineae* infections are prevalent. However, domestic cases in patients without travel history are increasingly being documented. The use of topical corticosteroid products often for severe pruritus can exacerbate the condition by masking symptoms and altering lesion morphology, complicating diagnosis and treatment (58, 68, 69). *T. indotineae* has rarely been implicated in nail or hair infections. To date, only two cases of onychomycosis have been reported—one from Italy in a patient who immigrated from India and the other in a French man—both involving toenails and associated with extensive tinea corporis, presenting with proximal erosion of the nail plate (70, 71). The reasons for the limited involvement of *T. indotineae* in hair and nail infections as compared to other *Trichophyton* species remain unknown. Pavlovic et al. (72) have suggested that poor keratolytic activity of *T. indotineae* may be the reason for rare nail infections. Some other possibilities may include its lack of lipolytic activity (1) or it may have just been underreported.

## ANTIFUNGAL DRUG RESISTANCE IN *T. INDOTINEAE*

Studies have shown limited effectiveness of first-line antifungals, namely, fluconazole, griseofulvin, TRB, and itraconazole, in treating dermatophytosis caused by *T. indotineae* (73). Isolates with TRB MICs of 0.5 µg/mL or higher are linked to treatment failure at standard doses and durations (12, 16, 26, 46, 74–76). These isolates often carry specific SQLE mutations, such as L393S and F397L (46) (Table 1). While increasing TRB doses to 500 mg daily may overcome mildly elevated MICs, response rates remain inconsistent. Isolates with SQLE mutations at position A448T are more likely to respond to TRB (77). However, Ebert et al. (12) reported that this mutation in the SQLE gene was present in both susceptible and resistant TRB isolates and was associated with increased MICs to itraconazole and voriconazole. The absence of established clinical breakpoints for dermatophytes and the limited correlation between *in vitro* antifungal susceptibility testing (AFST) and clinical outcomes pose significant challenges (46). Alternative treatments showed variable success: fluconazole led to improvement in two of four cases and griseofulvin in two of five cases, and itraconazole cleared or improved infection in five of seven cases. However, some patients discontinued treatment or were lost to follow-up (78).

**TABLE 1:** Summary of antifungal resistance genes, mutations, and associated phenotypes in *T. indotineae*

Antifungal resistance gene	Encoded protein	Mutations	Phenotypic resistance	Reference
*erg1*	SQLE	^*a*^L393S, ^*a*^L393F, ^*a*^F397L, F397I, ^*b*^A448T, Q408L, A392S, D352N, E174D, T52A, V24A, F415S, H440Y, F484Y, I121M, V237I, P397L, L393P, F397L/A448T, L393S/A448T	TRB	(12, 14, 24, 46, 58–60, 79–82)
		A448T, F397L/A448T	Azoles	(58, 74)
*erg11*	Lanosterol 14-α demethylase	G443E, Y444H, Y444C, A230T/D441G, A230T/Y444H	Azoles	(83)
*TinCYP51B*	Sterol 14α-demethylase	Gene duplication (Types I and II)		(14, 61, 84)

^*a*^ Most frequently reported mutations.

^*b*^ This amino acid substitution has been found in both TRB-resistant and susceptible isolates (12).

Mutations in the SQLE gene are well-established contributors to antifungal resistance in *T. indotineae* (2). However, azole resistance has also been recently identified in strains exhibiting upregulation of ABC transporters or mutations in the *erg11/CYP51* gene. The *erg11* gene encodes the lanosterol 14-α-demethylase enzyme, a key component in the ergosterol biosynthesis pathway targeted by azoles (61, 79).

In Canada, 47 clinical cases of *T. indotineae* were identified in Ontario from 2014 to 2023. Among 50 isolates analyzed, 71.4% were TRB-resistant, primarily due to L393F or L393S or F397L mutations. In 23.7% of isolates, decreased susceptibility to itraconazole or voriconazole was noted, which was frequently linked to *CYP51B* gene duplication (14).

*T. indotineae* strains in Germany, dating back to 2011, were found to have reduced *in vitro* susceptibility to itraconazole. This resistance was linked to the c.1342G>A SQLE gene mutation; however, it has not been confirmed to what extent this mutation contributes to the therapeutic failure of itraconazole *in vivo* (3). Burmester et al. (74) concluded that resistance against TRB and azoles develops independently over time, making antifungal resistance gene analysis important. Relying on species identification alone to predict response to treatment is not adequate.

## DIAGNOSIS AND CHALLENGES IN THE LABORATORY DETECTION OF *T. INDOTINEAE*

Clinical diagnosis of tinea infections is challenging due to their resemblance to other conditions with similar lesions. For instance, eczema can mimic tinea corporis, and alopecia areata can mimic tinea capitis (TC), and nail dystrophy caused by repeated minor trauma can mimic onychomycosis (85, 86). Both resemblance to other lesions and alteration in the appearance of the lesion due to prior treatment with steroids and antifungal agents can lead to misdiagnosis. Accurate diagnosis of dermatophytosis is essential for timely treatment and preventing transmission.

### Microscopy and culture

While the gold standard for dermatophyte identification remains microscopic examination of clinical samples, followed by culture to determine the specific fungal species (86, 87), these methods cannot reliably identify *T. indotineae*. Morphological identification is particularly challenging due to overlapping microscopic and macroscopic features between *T. indotineae* and its related species *T. mentagrophytes/T. interdigitale* (3, 63).

Physiological tests such as the urea slant test and HPT are helpful in differentiating *T. mentagrophytes* from *T. indotineae*. However, these tests require prolonged incubation, are not routinely performed in routine mycology laboratories, and cannot decisively identify to the species level (63, 83). Overall, the culture method and subsequent identification process are quite lengthy, making it unsuitable as a routine diagnostic test for dermatophytosis caused by *T. indotineae*.

### Matrix-assisted laser desorption/ionization time-of-flight mass-spectrometry

Matrix-assisted laser desorption/ionization time-of-flight mass-spectrometry (MALDI-TOF MS) has emerged as a fast and accurate method for identifying fungal species, often preferred over traditional morphology-based identification, which is time-consuming and requires skilled personnel. The commonly used MALDI-TOF MS system for fungal detection is the Bruker Biotyper platform along with its commercial identification library (88). However, the rates of species-level identification of dermatophytes using this system alone are low. In one study, it ranged only from 0% to 23.8% (88). Identification can be improved by including in-house libraries. Tsai et al. (88) showed that species-level identification of *Trichophyton* species improved to 90.7% by including an in-house library as compared to 16.1% by the Bruker library. In this study, none of the 10 *T. indotineae* isolates identified using the expanded library (Bruker + In-house) were identified using the Bruker library alone, consistent with the fact that *T. indotineae* is not yet included in the Bruker database (MBT Filamentous Fungi Library 2023 release notes, Bruker). Another study evaluating an in-house fungal MS library successfully identified 20 *T. indotineae* strains at the species level, even in the presence of reference spectra for *T. mentagrophytes* and *T. interdigitale* (89).

Recent advancements, including the online MSI-2 application, have demonstrated the capability of MALDI-TOF MS to accurately identify *T. indotineae*. MSI-2 is an improved version of MSI-1, which was developed in 2019 by Sorbonne University (Paris, France) and is available at https://msi.happy-dev.fr/. It is an online mass spectrum identification application coded in Python (90). When in-house databases fall short, the free improved MSI V2.0 tool offers a reliable alternative (56), achieving 100% identification accuracy for *T. indotineae* strains, with 95% being considered highly reliable for identification to the species level (89). Normand et al*.* described two peaks, 6,845 and 10,680 Da, to be the most discriminating between *T. indotineae* and *T. mentagrophytes/interdigitale* in colonies that are 7 to 10 days old (56). As of now, the use of MSI applications for detecting *T. indotineae* from MALDI-TOF MS spectra obtained from fungal isolates has only been described for Bruker systems (56, 90).

While specialized MALDI-TOF MS databases enable the identification of *T. indotineae*, the process relies on obtaining a positive culture with a mature isolate, potentially delaying the diagnosis by 1–2 weeks. Moreover, MALDI-TOF MS currently cannot distinguish between TRB-resistant and TRB-susceptible strains. The inability to link resistance to specific reference spectra may stem from the limitations of MALDI-TOF technology, as TRB resistance often results from single mutations in the SQLE gene (89). Future enhancements in specificity and detection might allow MALDI-TOF MS to combine species identification and resistance screening in one step. Promising approaches include comparing mass spectra of fungi incubated with antifungal drugs or using the MALDI Biotyper Antibiotic Susceptibility Test Rapid Assay. The latter, a phenotypic assay measuring fungal growth via mass spectra, has shown success in detecting resistance in *Candida* species. While MALDI-TOF MS reliably identifies *T. indotineae*, advancements are needed to detect antifungal resistance, particularly in dermatophytes (56, 89).

### ITS sequencing

ITS sequencing of *T. indotineae* was first described by Kano et al*.* (10). This played a key role in designating this organism as a distinct *Trichophyton* species. In their study, ITS sequencing of two highly TRB-resistant *T. interdigitale*-like strains from a Nepali patient and an Indian patient with tinea corporis revealed 99.5% similarity with previously reported *T. interdigitale* isolates, suggesting that they fell within intra-species genetic variation (10). However, phylogenetic analysis demonstrated that these strains formed a distinct cluster associated with Indian strains, separate from the *T. interdigitale* cluster. Singh et al. (16) further established that Indian strains are genetically unique compared to global reference strains. In the ITS region, the two *T. indotineae* isolates, along with all Indian TRB-resistant *T. interdigitale* isolates, exhibited three SNPs at positions 94 (C), 125 (T), and 462 (T).

Since these ITS region differences were first described, ITS sequencing has been widely adopted as a reference method for confirming the identification of *T. indotineae* in culture. However, ITS sequencing is a cumbersome process, as it involves multiple steps, including DNA extraction, amplification of the ITS region using universal primers, purification of the polymerase chain reaction (PCR) products, and DNA sequencing. Furthermore, many diagnostic laboratories lack the expertise and equipment necessary for sequencing, limiting the utility of ITS sequencing as a routine diagnostic method for detecting *T. indotineae*. Other than ITS sequencing, molecular assays for the detection of *T. indotineae* are limited but emerging.

### Polymerase chain reaction

PCR has become increasingly popular for diagnosing dermatophytes directly from the specimen. Batvandi et al*.* introduced two cost-effective PCR-based assays to differentiate *T. indotineae* from other dermatophytes. A PCR-restriction fragment length polymorphism assay targeting TOP2 sequences and a species-specific endpoint PCR for the C120-287 locus validated on 193 strains including 41 *T. indotineae.* Both assays demonstrated 99% specificity (91). However, these assays were not applied to DNA extracts from clinical specimens. Therefore, the sensitivity and specificity of the assays as routine diagnostic tools remain unknown. Furthermore, the workflow is cumbersome, and there are risks of contamination during testing because these assays rely on traditional gel electrophoresis techniques (91)

Recently, Baron et al. described a dual quantitative PCR assay for the rapid detection of *T. indotineae* from clinical samples (92). This assay targets a single specific polymorphism in the ITS region. While it successfully differentiated *T. indotineae* from several non-dermatophyte and dermatophyte species, it weakly detected some other species within the TMC. Consequently, the study proposed a Cq value-based approach to specifically detect and differentiate *T. indotineae* from other TMC species (92, 93). While this study demonstrates the potential utility of a quantitative real-time PCR assay for detecting *T. indotineae* directly in DNA extracts from clinical samples offering a rapid turnaround time (TAT), further optimization of the test design may be necessary to ensure high specificity (94).

### Antifungal susceptibility test

In 2017, Yamada et al. (95) reported TRB resistance in 17 clinical isolates of *T. rubrum* and *T. interdigitale* that showed reduced TRB susceptibility. Resistance to TRB was linked to mutations in the SQLE gene, with hotspots at amino acid positions L393, F397, F415, and H440. The most frequent substitutions, F397L and L393F, are associated with high TRB MICs and accounted for resistance in two-thirds of global studies (94). Despite the rising prevalence of dermatophyte infections failing TRB therapy, AFST is not routinely performed for dermatophytes, and no breakpoints classifying isolates as susceptible or resistant are available. This makes interpreting the clinical impact of minimal inhibitory concentration (MIC) difficult, leading to underestimation of resistance rates (96–99).

The EUCAST E.Def 11.0 method, developed by the European Committee on Antimicrobial Susceptibility Testing, provides a standardized approach for AFST of dermatophytes (97). It is specifically designed for *T. rubrum* and *T. interdigitale* and includes modifications such as a dermatophyte-specific medium, adjusted incubation parameters, and spectrophotometric growth measurement. This method is validated for TRB, voriconazole, itraconazole, and amorolfine and can be integrated into the existing EUCAST workflows (97, 98). A 2020 multicenter study validated this method and established wild-type upper MIC limits (WT-UL) for these species (97). However, subsequent taxonomic revisions introduced *T. indotineae* as a distinct species, predominantly found in TRB-resistant Indian isolates. As a result, *T. indotineae* has been documented in the literature under all three species names, and susceptibility data for the species complex cannot be accurately attributed to individual species without molecular identification (96). Recent studies suggest wild-type *T. indotineae* exhibits slightly higher MICs compared to wild-type *T. interdigitale* isolates, indicating potential differences in their epidemiological cutoff values (62, 80). Using the EUCAST E.Def 11.0 method, Siopi et al. (80) reported *T. interdigitale* isolates had modal MICs two-fold dilution lower than susceptible *T. indotineae*. Similarly, Kong *et al*. found TRB modal MICs of 0.016 mg/L for *T. interdigitale* and 0.06 mg/L for *T. indotineae* using the EUCAST 9.3 mold method (62). These findings confirm that previously established WT-UL values (TRB: 0.125 mg/L; voriconazole: 1 mg/L; itraconazole: 0.25 mg/L; amorolfine: 0.5 mg/L) apply to *T. indotineae* rather than *T. interdigitale* (96). Molecular identification is therefore essential to accurately link susceptibility data to specific species within this complex (96–98). The EUCAST E.Def.11.0 microdilution method, though standard, is labor-intensive and requires specialized training (100).

TRB gradient strips (HiMedia, PA, USA) are specialized MIC determination tools coated with TRB in a concentration gradient, capable of measuring MICs from 0.002 to 32 µg/mL when tested against target organisms (100). Pure cultures should be obtained, and dermatophytes should be cultured on PDA or Oatmeal Agar at 30°C for 4–5 days or until substantial conidial growth is observed (100). Recent evaluations compared MIC values for 79 *Trichophyton* spp*.* isolates determined by TRB gradient strips and the EUCAST reference method. Gradient strips showed lower MICs (MIC_50_: 0.002µg/mL) compared to EUCAST (MIC50: 0.016  µg/mL). While categorical agreement between the methods exceeded 90%, essential agreement varied across gradient strip batches (100).

Earlier studies focused on detecting TRB resistance through PCR targeting hotspot mutation sites in the SQLE genes of dermatophytes. One such assay is the commercial DermaGenius Resistance Multiplex real-time PCR, which detects L393F, F397L, L393S, F397I, and F397V mutations in the SQLE gene (94). The DermaGenius Resistance real-time PCR assay offers a rapid alternative, detecting common SQLE mutations linked to high TRB resistance. In a study evaluating 97 *Trichophyton* isolates, the assay accurately identified all TRB-resistant strains with these mutations, showing perfect agreement with AFST and sequencing results (94). The PCR kit can directly test clinical samples, significantly reducing TAT compared to conventional methods, which can take over a month. Limitations include the assay’s inability to specify mutations, as it only identifies a mutant or wild-type profile without determining amino acid substitutions in SQLE. Although the assay targets the most common SQLE mutations, reliance on predefined mutation targets may lead to false-negative results when antifungal resistance is associated with rare or emerging mutations. Additionally, the high capital cost of real-time thermocyclers and the high operating costs may limit their accessibility in resource-limited settings (24, 75, 94).

A recent study assessed TRB resistance in 28 *Trichophyton* strains using gradient strips, EUCAST E.Def 11.0 microdilution, DermaGenius Resistance PCR assay, and SQLE sequencing, with WGS as the reference method (75). All methods successfully identified resistant strains through elevated MICs (>0.125  µg/mL) or SQLE mutations (75). Gradient strips showed good categorical agreement with EUCAST but variable essential agreement (±2 log2). Despite batch variability, they were cost-effective and suitable for initial resistance screening in low-resource settings. However, confirmatory testing using EUCAST E.Def 11.0 remains essential (75). The DermaGenius PCR assay demonstrated perfect agreement with EUCAST, gradient strips, and WGS in detecting common SQLE mutations (F397L and L393F) linked to high TRB MICs. Its rapid processing reduces TAT and improves patient management (24, 75).

## TREATMENT OPTIONS AND CHALLENGES IN MANAGING DERMATOPHYTOSIS CAUSED BY *T. INDOTINEAE*

The primary treatment for dermatomycoses typically involves topical antifungals. The main classes of topical antifungal agents include azoles such as clotrimazole and allylamines such as TRB and benzylamines such as butenafine (101, 102). When topical therapy fails, oral antifungals such as oral TRB, itraconazole, ketoconazole, or fluconazole are commonly prescribed (103). Griseofulvin is an alternative but requires a longer duration of treatment and carries a risk of drug interactions. Combined regimens of topical and oral antifungals with anti-inflammatory agents have also been used to enhance cure rates (104).

However, many chronic and recurrent dermatophytosis cases, particularly those caused by *T. indotineae*, are unresponsive to topical or oral TRB due to its high resistance. A daily dose of oral TRB, 250 mg for 4–8 weeks, is often prescribed as first-line treatment and achieves clinical cure in patients with TRB-susceptible strains of *T. indotineae* (26, 105). Increasing the dosage to 250 mg twice daily in cases of treatment failure with standard dosing has shown some success, although the failure rate remains significant at 30% (13, 106). Itraconazole is the preferred treatment for *T. indotineae* infections with a recommended dose of 100 mg twice daily for 4–8 weeks, extendable to 12 weeks for some cases (3). Sonego et al*.* described itraconazole 200 mg per day for a variable duration of 1 to 12 weeks as the most effective treatment for *T. indotineae* infections. Super bioavailability (SUBA)-itraconazole has shown efficacy at a dose of 50 mg twice daily for 4-8 weeks (3, 107). This is due to increased bioavailability. Furthermore, serum levels of SUBA are more stable, and the need for an acidic environment is not required (81). While fluconazole and griseofulvin are options, they often exhibit increased MICs against *T. indotineae*, and clinical responses vary (3, 81). In a study from Banaras Hindu University in India, treatment with fluconazole, griseofulvin, TRB, and itraconazole showed limited effectiveness at 4 weeks, with cure rates below 8%. By eight weeks, itraconazole demonstrated the highest cure rate (66%), outperforming fluconazole (42%), TRB (28%), and griseofulvin (14%) (3, 73, 108).

Voriconazole has shown success in treating multidrug-resistant *T. indotineae* infections, including familial cases, with good efficacy and a low relapse rate. However, its use should be limited to confirmed multidrug-resistant cases to avoid resistance development. Combination antifungal therapies are being explored, though further studies on safety and efficacy are needed. Topical antifungals, including azoles, TRB, and ciclopirox, can be used with systemic treatments or alone in vulnerable populations like infants, pregnant women, or those with comorbidities (27).

A recent review of 25 articles involving 58 patients with tinea caused by *T. indotineae*, confirmed via molecular analysis, concludes that systemic azoles should remain the cornerstone of therapy, with oral itraconazole (≥200 mg/day) recommended as a first-line option, even for cases involving SQLE mutations like L393S and F397L (81). Oral voriconazole, though effective in some cases, is not recommended as routine therapy due to its critical role in managing invasive fungal infections. TRB (250 mg/day) may still be effective for wild-type or A448T SQLE-mutated strains, but rising resistance limits its reliability. While griseofulvin and fluconazole have not shown much success, Caplan et al*.* argue that griseofulvin may be a better option to itraconazole, considering the challenges with pharmacokinetics and adverse events. They have shown that griseofulvin was effective in treating their patients, although some patients may require longer durations and higher doses requiring close monitoring (109). Systemic combination therapies, including antifungal combinations or their use with retinoids, require further research to confirm their efficacy. AFST combined with molecular analysis of SQLE mutations is essential for guiding treatment decisions. However, emerging strains of *T. indotineae* with double mutations in SQLE and *erg11* present significant therapeutic challenges due to reduced sensitivity to both allylamines and azoles. This underscores the urgent need for optimized treatment strategies and the development of new antifungal agents to address the growing threat of *T. indotineae* infections (81).

Most cases where topical treatment has shown efficacy are when used in combination with systemic antifungal agents (81). Only a limited number of cases have been described in the literature where topical antifungal agents alone were successful in treating these infections (26, 46, 110).

## PERSPECTIVES

The emergence of *T. indotineae*, a novel and highly TRB-resistant dermatophyte, represents a global health challenge. Despite growing global reports, epidemiological studies on the incidence and prevalence of *T. indotineae* remain limited. Its recent spread to regions like the USA, Canada, Europe, and Argentina highlights the global impact of this emerging dermatophyte. Diagnostic challenges with *T. indotineae* are multifaceted. Diagnostic testing to confirm suspected dermatophytosis is rarely requested by clinicians, with AFST being even less commonly ordered. An observational cohort study of 3.9 million US children found that confirmatory diagnostic testing was performed in only 21.9% of cases of TC, primarily using fungal culture (17.8%) or direct microscopy (9.7%) (111). In high-risk patients such as individuals belonging to countries endemic for *T. indotineae* or travel history to countries with high incidence of *T. indotineae* and in patients presenting with extensive dermatophytosis not responding to standard therapy, *T. indotineae* should be high on the differential. Laboratory diagnosis and AFST should be performed on these patients to establish the diagnosis and to provide appropriate therapy, especially in countries with low prevalence, to prevent further spread of the infection.

Barriers to testing include low reimbursement rates, lengthy TATs, and limited access to laboratories capable of species-level identification and AFST. Furthermore, the absence of FDA-approved testing platforms and established clinical resistance breakpoints complicates the process. Dermatophytosis, including antifungal-resistant cases, is not classified as a nationally notifiable condition in many countries, including the United States. This also relates to the lack of knowledge regarding the actual burden of *T. indotineae*-mediated dermatophytosis. It is likely that a large number of *T. indotineae* cases may have gone undetected because of the lack of testing or the lack of testing to species-level resolution. This notion is supported by the recent report from the United Kingdom showing 38% of dermatophytes tested at a referral laboratory were positive for *T. indotineae*, while up to 78% of patients with dermatophytosis in India were tested positive for *T. indotineae* about 5 years ago (63).

Considering the widespread emergence of antifungal-resistant *T. indotineae*, there is an urgent need for improved laboratory detection methods for diagnosis and surveillance. Given the long TAT of culture-based identification methods coupled with its phenotypic similarity to *T. mentagrophytes* and *T. interdigitale*, molecular biology remains the most appropriate approach for identifying *T. indotineae*. While MALDI-TOF offers inexpensive and rapid identification, the requirement for fungal culture growth persists. Techniques such as ITS region sequencing provide reliable differentiation but are time-consuming, cumbersome, and costly. Furthermore, no studies have yet evaluated whether ITS sequencing can be applied directly to clinical specimens.

A suitable probe-based qPCR assay capable of direct application to clinical samples without requiring culture appears to be the most practical option for the rapid detection of *T. indotineae* and antifungal resistance. However, challenges persist in designing PCR assays due to genomic sequence homology with *T. interdigitale* and *T. mentagrophytes*, as well as in optimizing fungal DNA extraction to achieve higher sensitivity. Large-scale WGS is therefore essential to create an extensive data set for genome alignment and the development of specific probe-based PCR assays targeting parts of the genome that are conserved among *T. indotineae* species isolated from different countries but are divergent enough to differentiate *T. indotineae* species from other closely related *Trichophyton* species. Additionally, optimizing sample pre-processing and DNA extraction from clinical samples, along with developing real-time, multiplex PCR assays for pan-dermatophyte detection, antifungal resistance, and *T. indotineae*, with a TAT of less than 24 hours, is crucial for the effective antifungal management of patients with tinea infections caused by *T. indotineae*. The development of a qPCR assay for the definitive identification of *T. indotineae* directly from clinical samples with superior performance characteristics will support large-scale screening programs. These programs can identify positive specimens for culture and facilitate the isolation of *T. indotineae* strains for genomic surveillance through WGS.

The integration of AFST with molecular testing for resistance markers, such as SQLE and *erg11* mutations, has been proposed in earlier studies (81). However, the lengthy TAT for AFSTs limits their utility in real-time patient management. Consequently, it is essential to extensively validate the performance characteristics of molecular identification and antifungal resistance assays against standard culture, identification, and phenotypic susceptibility methods to achieve acceptable sensitivity and specificity of the test.

Managing dermatophytosis caused by *T. indotineae* presents significant treatment challenges due to the pathogen’s increasing resistance to first-line antifungal agents such as TRB and griseofulvin. Effective treatment options are limited, highlighting the need for standardized trials and optimized regimens. Currently, itraconazole is considered the drug of choice. Although voriconazole has been shown to be effective, it is reserved for managing systemic fungal infections. Combination therapies, such as combining antifungals with anti-inflammatory agents or retinoids, are under investigation and require further study. In addition to pharmacologic treatment, good hygiene practices are crucial for preventing transmission and reinfection. Standardized clinical trials are urgently needed to establish treatment guidelines, particularly for combination therapy, given the potential for multidrug resistance in *T. indotineae* (79).

A rapid, reliable laboratory test for *T. indotineae* is crucial not only for effective patient management but also for antifungal stewardship. With limited treatment options, robust research in the development of new treatment strategies and evidence-based treatment guidelines for *T. indotineae* are urgently needed. In addition, robust public health measures such as surveillance programs, enhancing clinician awareness by providing guidance on prevention, diagnosis and treatment, and improving access to testing are needed to prevent the spread, and to facilitate diagnosis and treatment of *T. indotineae* dermatophytosis.

## References

[1] Tang C, Kong X, Ahmed SA, Thakur R, Chowdhary A, Nenoff P, Uhrlass S, Verma SB, Meis JF, Kandemir H, Kang Y, de Hoog GS. 2021. Taxonomy of the Trichophyton mentagrophytes/T. interdigitale species complex harboring the highly virulent, multiresistant genotype T. indotineae. Mycopathologia 186:315–326. doi:10.1007/s11046-021-00544-2 33847867 PMC8249266

[2] Chowdhary A, Singh A, Kaur A, Khurana A. 2022. The emergence and worldwide spread of the species Trichophyton indotineae causing difficult-to-treat dermatophytosis: a new challenge in the management of dermatophytosis. PLoS Pathog 18:e1010795. doi:10.1371/journal.ppat.1010795 36173977 PMC9521800

[3] Uhrlaß S, Verma SB, Gräser Y, Rezaei-Matehkolaei A, Hatami M, Schaller M, Nenoff P. 2022. Trichophyton indotineae-an emerging pathogen causing recalcitrant dermatophytoses in India and worldwide-a multidimensional perspective. J Fungi (Basel) 8:757. doi:10.3390/jof8070757 35887512 PMC9323571

[4] Dellière S, Jabet A, Abdolrasouli A. 2024. Current and emerging issues in dermatophyte infections. PLoS Pathog 20:e1012258. doi:10.1371/journal.ppat.1012258 38870096 PMC11175395

[5] Rand S. 2000. Overview: the treatment of dermatophytosis. J Am Acad Dermatol 43:S104–S112. doi:10.1067/mjd.2000.110380 11044285

[6] Dogra S, Uprety S. 2016. The menace of chronic and recurrent dermatophytosis in India: Is the problem deeper than we perceive? Indian Dermatol Online J 7:73–76. doi:10.4103/2229-5178.178100 27057485 PMC4804598

[7] Verma S, Madhu R. 2017. The great indian epidemic of superficial dermatophytosis: an appraisal. Indian J Dermatol 62:227–236. doi:10.4103/ijd.IJD_206_17 28584364 PMC5448256

[8] Bhattacharya T, Datta J, Sen I, Patra AC, Roy S, Sarkar AP, Das NK. 2022. Perception among the sufferers of recalcitrant dermatophytosis regarding its causation, prevention, care-seeking behaviour and their personal hygiene: a qualitative research. Indian Dermatol Online J 13:52–59. doi:10.4103/idoj.idoj_211_21 35198468 PMC8809170

[9] Nenoff P, Verma SB, Vasani R, Burmester A, Hipler UC, Wittig F, Krüger C, Nenoff K, Wiegand C, Saraswat A, Madhu R, Panda S, Das A, Kura M, Jain A, Koch D, Gräser Y, Uhrlaß S. 2019. The current Indian epidemic of superficial dermatophytosis due to Trichophyton mentagrophytes-a molecular study. Mycoses 62:336–356. doi:10.1111/myc.12878 30561859

[10] Kano R, Kimura U, Kakurai M, Hiruma J, Kamata H, Suga Y, Harada K. 2020. Trichophyton indotineae sp. nov.: a new highly terbinafine-resistant anthropophilic dermatophyte species. Mycopathologia 185:947–958. doi:10.1007/s11046-020-00455-8 32449054

[11] Verma SB, Khurana A, Bosshard PP, Kargl A, Singal A, Saraswat A, Guenova E, Schaller M, Panda S, Rezaei-Matehkolaei A, et al.. 2025. “Trichophyton indotineae” is an inaccurate and pejorative term. Indian J Dermatol Venereol Leprol 91:277–280. doi:10.25259/IJDVL_1793_2024 39912159

[12] Ebert A, Monod M, Salamin K, Burmester A, Uhrlaß S, Wiegand C, Hipler UC, Krüger C, Koch D, Wittig F, Verma SB, Singal A, Gupta S, Vasani R, Saraswat A, Madhu R, Panda S, Das A, Kura MM, Kumar A, Poojary S, Schirm S, Gräser Y, Paasch U, Nenoff P. 2020. Alarming India-wide phenomenon of antifungal resistance in dermatophytes: a multicentre study. Mycoses 63:717–728. doi:10.1111/myc.13091 32301159

[13] Jabet A, Normand A-C, Brun S, Dannaoui E, Bachmeyer C, Piarroux R, Hennequin C, Moreno-Sabater A. 2023. Trichophyton indotineae, from epidemiology to therapeutic. J Med Mycol 33:101383. doi:10.1016/j.mycmed.2023.101383 37031652

[14] McTaggart LR, Cronin K, Ruscica S, Patel SN, Kus JV. 2025. Emergence of terbinafine-resistant Trichophyton indotineae in Ontario, Canada, 2014-2023. J Clin Microbiol 63:e0153524. doi:10.1128/jcm.01535-24 39584838 PMC11784349

[15] Singh A, Masih A, Khurana A, Singh PK, Gupta M, Hagen F, Meis JF, Chowdhary A. 2018. High terbinafine resistance in Trichophyton interdigitale isolates in Delhi, India harbouring mutations in the squalene epoxidase gene . Mycoses 61:477–484. doi:10.1111/myc.12772 29577447

[16] Singh A, Masih A, Monroy-Nieto J, Singh PK, Bowers J, Travis J, Khurana A, Engelthaler DM, Meis JF, Chowdhary A. 2019. A unique multidrug-resistant clonal Trichophyton population distinct from Trichophyton mentagrophytes/Trichophyton interdigitale complex causing an ongoing alarming dermatophytosis outbreak in India: Genomic insights and resistance profile. Fungal Genet Biol 133:103266. doi:10.1016/j.fgb.2019.103266 31491507

[17] Nenoff P, Verma SB, Uhrlaß S, Burmester A, Gräser Y. 2019. A clarion call for preventing taxonomical errors of dermatophytes using the example of the novel Trichophyton mentagrophytes genotype VIII uniformly isolated in the Indian epidemic of superficial dermatophytosis . Mycoses 62:6–10. doi:10.1111/myc.12848 30187579

[18] Verma SB, Vasani R. 2016. Male genital dermatophytosis - clinical features and the effects of the misuse of topical steroids and steroid combinations - an alarming problem in India. Mycoses 59:606–614. doi:10.1111/myc.12503 27028087

[19] Verma SB, Zouboulis C. 2018. Indian irrational skin creams and steroid-modified dermatophytosis - an unholy nexus and alarming situation. J Eur Acad Dermatol Venereol 32:e426–e427. doi:10.1111/jdv.15025 29706004

[20] Sheth NK, Nair PA. 2021. Topical steroids: awareness and misuse among patients, pharmacists and general medical practitioner. Indian J Dermatol Venereol Leprol 87:54–59. doi:10.4103/ijdvl.IJDVL_84_18 30971536

[21] Singal A, Jakhar D, Kaur I, Pandhi D, Das S. 2019. Tinea pseudoimbricata as a unique manifestation of steroid abuse: a clinico-mycological and dermoscopic study from a tertiary care hospital. Indian Dermatol Online J 10:422. doi:10.4103/idoj.IDOJ_385_18 31334062 PMC6615389

[22] Thakran P, Agrawal S, Singal A, Verma S, Madhu SV. 2021. Iatrogenic cushing's syndrome in patients with superficial dermatophytosis. Indian Dermatol Online J 12:237–243. doi:10.4103/idoj.IDOJ_432_20 33959519 PMC8088179

[23] Sardana K, Gupta A, Mathachan SR. 2021. Immunopathogenesis of dermatophytoses and factors leading to recalcitrant infections. Indian Dermatol Online J 12:389–399. doi:10.4103/idoj.IDOJ_503_20 34211904 PMC8202482

[24] Bhuiyan MSI, Verma SB, Illigner GM, Uhrlaß S, Klonowski E, Burmester A, Noor T, Nenoff P. 2024. Trichophyton mentagrophytes ITS genotype VIII/Trichophyton indotineae infection and antifungal resistance in Bangladesh. J Fungi (Basel) 10:768. doi:10.3390/jof10110768 39590687 PMC11595601

[25] Kawasaki M, Anzawa K, Wakasa A, Takeda K, Tanabe H, Mochizuki T, Ishizaki H, M.Hemashettar B. 2008. Different genes can result in different phylogenetic relationships in trichophyton species. Nippon Ishinkin Gakkai Zasshi 49:311–318. doi:10.3314/jjmm.49.311 19001759

[26] Jabet A, Brun S, Normand AC, Imbert S, Akhoundi M, Dannaoui E, Audiffred L, Chasset F, Izri A, Laroche L, Piarroux R, Bachmeyer C, Hennequin C, Sabater AM. 2022. Extensive dermatophytosis caused by terbinafine-resistant Trichophyton indotineae, France. Emerg Infect Dis 28:229–233. doi:10.3201/eid2801.210883 34932456 PMC8714191

[27] Jia S, Long X, Hu W, Zhu J, Jiang Y, Ahmed S, de Hoog GS, Liu W, Jiang Y. 2022. The epidemic of the multiresistant dermatophyte Trichophyton indotineae has reached China. Front Immunol 13:1113065. doi:10.3389/fimmu.2022.1113065 36874152 PMC9978415

[28] Abdolrasouli A, Hay RJ. 2024. Antifungal-resistant Trichophyton indotineae: transmission is occurring outside previously identified endemic areas - are we prepared? Br J Dermatol 191:145–146. doi:10.1093/bjd/ljae140 38593243

[29] Taghipour S, Shamsizadeh F, Pchelin IM, Rezaei-Matehhkolaei A, Zarei Mahmoudabadi A, Valadan R, Ansari S, Katiraee F, Pakshir K, Zomorodian K, Abastabar M. 2020. Emergence of terbinafine resistant Trichophyton mentagrophytes in Iran, harboring mutations in the squalene epoxidase (SQLE) gene. Infect Drug Resist 13:845–850. doi:10.2147/IDR.S246025 32214830 PMC7078656

[30] Haghani I, Babaie M, Hoseinnejad A, Rezaei-Matehkolaei A, Mofarrah R, Yahyazadeh Z, Kermani F, Javidnia J, Shokohi T, Azish M, Kamyab Hesari K, Saeedi M, Ghasemi Z, Khojasteh S, Hajheydari Z, Mosayebi E, Valadan R, Seyedmousavi S, Abastabar M, Hedayati MT. 2024. High prevalence of terbinafine resistance among Trichophyton mentagrophytes/T. interdigitale species complex, a cross-sectional study from 2021 to 2022 in northern parts of Iran. Mycopathologia 189:52. doi:10.1007/s11046-024-00855-0 38864945

[31] Ngo TMC, Ton Nu PA, Le CC, Ha TNT, Do TBT, Tran Thi G. 2022. First detection of Trichophyton indotineae causing tinea corporis in Central Vietnam. Med Mycol Case Rep 36:37–41. doi:10.1016/j.mmcr.2022.05.004 35620657 PMC9127533

[32] Süß A, Uhrlaß S, Ludes A, Verma SB, Monod M, Krüger C, Nenoff P. 2019. Ausgeprägte Tinea corporis durch ein Terbinafin-resistentes Trichophyton-mentagrophytes-Isolat vom indischen Genotyp bei einem Säugling aus Bahrain in Deutschland. Hautarzt 70:888–896. doi:10.1007/s00105-019-4431-7 31098692

[33] Saunte DML, Pereiro‐Ferreirós M, Rodríguez‐Cerdeira C, Sergeev AY, Arabatzis M, Prohić A, Piraccini BM, Lecerf P, Nenoff P, Kotrekhova LP, Bosshard PP, Padovese V, Szepietowski JC, Sigurgeirsson B, Nowicki RJ, Schmid‐Grendelmeier P, Hay RJ. 2021. Emerging antifungal treatment failure of dermatophytosis in Europe: take care or it may become endemic. Acad Dermatol Venereol 35:1582–1586. doi:10.1111/jdv.17241 33768571

[34] Tan TY, Wang YS, Wong XYA, Rajandran P, Tan MG, Tan AL, Tan YE. 2024. First reported case of Trichophyton indotineae dermatophytosis in Singapore. Pathology (Phila) 56:909–913. doi:10.1016/j.pathol.2024.04.003 38937185

[35] Madarasingha NP, Thabrew H, Uhrlass S, Eriyagama S, Reinal D, Jayasekera PI, Nenoff P. 2024. Dermatophytosis caused by Trichophyton indotineae (Trichophyton mentagrophytes ITS Genotype VIII) in Sri Lanka. Am J Trop Med Hyg 111:575–577. doi:10.4269/ajtmh.24-0027 38981494 PMC11376159

[36] Posso-De Los Rios CJ, Tadros E, Summerbell RC, Scott JA. 2022. Terbinafine resistant Trichophyton indotineae isolated in patients with superficial dermatophyte infection in Canadian patients. J Cutan Med Surg 26:371–376. doi:10.1177/12034754221077891 35144480

[37] Brasch J, Gräser Y, Beck‐Jendroscheck V, Voss K, Torz K, Walther G, Schwarz T. 2021. “Indian” strains of Trichophyton mentagrophytes with reduced itraconazole susceptibility in Germany. J Deutsche Derma Gesell 19:1723–1727. doi:10.1111/ddg.14626 34850554

[38] Mohseni S, Abou-Chakra N, Oldberg K, Chryssanthou E, Young E. 2025. Terbinafine resistant Trichophyton indotineae in Sweden. Acta Derm Venereol 105:adv42089. doi:10.2340/actadv.v105.42089 39927724 PMC11833249

[39] Dashti Y, Alobaid K, Al-Rashidi S, Dashti M, AbdulMoneim MH, Al-Enezi M, Abou-Chakra N, Jørgensen KM. 2023. Autochthonous case of Trichophyton indotineae in Kuwait. J Mycol Med 33:101432. doi:10.1016/j.mycmed.2023.101432 37666031

[40] Durdu M, Kandemir H, Karakoyun AS, Ilkit M, Tang C, de Hoog S. 2023. First terbinafine-resistant Trichophyton indotineae isolates with Phe397Leu and/or Thr414His mutations in Turkey. Mycopathologia 188:295–304. doi:10.1007/s11046-023-00708-2 36656402

[41] Messina F, Santiso G, Romero M, Bonifaz A, Fernandez M, Marin E. 2023. First case report of tinea corporis caused by Trichophyton indotineae in Latin America. Med Mycol Case Rep 41:48–51. doi:10.1016/j.mmcr.2023.08.004 37706043 PMC10495376

[42] Mosam A, Shuping L, Naicker S, Maphanga T, Tsotetsi E, Mudau R, Maluleka C, Mpembe R, Ismail H, Singh A. 2023. A case of antifungal-resistant ringworm infection in KwaZulu-Natal Province, South Africa, caused by Trichophyton indotineae. Public Health Bulletin, South Africa.

[43] Guschin AE, Romanova IV, Ilyin LA, Potekaev NN. 2024. First description of cases of superficial mycoses caused by the resistant to allylamines (terbinafine) «Indian» species of dermatophytes — Trichophyton indotineae, in Russian dermatovenereological practice. Rus J Clin Derma Vener 23:581. doi:10.17116/klinderma202423051581

[44] Klinger M, Theiler M, Bosshard PP. 2021. Epidemiological and clinical aspects of Trichophyton mentagrophytes/Trichophyton interdigitale infections in the Zurich area: a retrospective study using genotyping. J Eur Acad Dermatol Venereol 35:1017–1025. doi:10.1111/jdv.17106 33411941

[45] Uhrlaß S MS, Koch D, Mütze H, Krüger C, Monod M, Nenoff P. 2024. Dermatophytes and skin dermatophytoses in Southeast Asia-First epidemiological survey from Cambodia. Mycoses 67:e13718. doi:10.1111/myc.13718 38551112

[46] Nenoff P, Verma SB, Ebert A, Süß A, Fischer E, Auerswald E, Dessoi S, Hofmann W, Schmidt S, Neubert K, et al.. 2020. Spread of terbinafine-resistant Trichophyton mentagrophytes type VIII (India) in Germany-"the Tip of the Iceberg?". J Fungi (Basel) 6:207. doi:10.3390/jof6040207 33027904 PMC7712673

[47] Saunte DML, Pereiro-Ferreirós M, Rodríguez-Cerdeira C, Sergeev AY, Arabatzis M, Prohić A, Piraccini BM, Lecerf P, Nenoff P, Kotrekhova LP, Bosshard PP, Padovese V, Szepietowski JC, Sigurgeirsson B, Nowicki RJ, Schmid-Grendelmeier P, Hay RJ. 2021. Emerging antifungal treatment failure of dermatophytosis in Europe: take care or it may become endemic. J Eur Acad Dermatol Venereol 35:1582–1586. doi:10.1111/jdv.17241 33768571

[48] Abdolrasouli A, Barton RC, Borman AM. 2025. Spread of antifungal-resistant Trichophyton indotineae, United Kingdom, 2017-2024. Emerg Infect Dis 31:192–194. doi:10.3201/eid3101.240923 39714510 PMC11682803

[49] Baert F, Stubbe D, D’hooge E, Packeu A, Hendrickx M. 2020. Updating the taxonomy of dermatophytes of the BCCM/IHEM collection according to the new standard: a phylogenetic approach. Mycopathologia 185:161–168. doi:10.1007/s11046-019-00338-7 31093849

[50] Cafarchia C, Weigl S, Figueredo LA, Otranto D. 2012. Molecular identification and phylogenesis of dermatophytes isolated from rabbit farms and rabbit farm workers. Vet Microbiol 154:395–402. doi:10.1016/j.vetmic.2011.07.021 21840652

[51] de Hoog GS, Dukik K, Monod M, Packeu A, Stubbe D, Hendrickx M, Kupsch C, Stielow JB, Freeke J, Göker M, Rezaei-Matehkolaei A, Mirhendi H, Gräser Y. 2017. Toward a novel multilocus phylogenetic taxonomy for the dermatophytes. Mycopathologia 182:5–31. doi:10.1007/s11046-016-0073-9 27783317 PMC5283515

[52] Makimura K, Mochizuki T, Hasegawa A, Uchida K, Saito H, Yamaguchi H. 1998. Phylogenetic classification of Trichophyton mentagrophytes complex strains based on DNA sequences of nuclear ribosomal internal transcribed spacer 1 regions. J Clin Microbiol 36:2629–2633. doi:10.1128/JCM.36.9.2629-2633.1998 9705405 PMC105175

[53] Moriello KA, Coyner K, Paterson S, Mignon B. 2017. Diagnosis and treatment of dermatophytosis in dogs and cats.: clinical consensus guidelines of the world association for veterinary dermatology. Vet Dermatol 28:266–e68. doi:10.1111/vde.12440 28516493

[54] Gräser Y, El Fari M, Vilgalys R, Kuijpers AF, De Hoog GS, Presber W, Tietz H. 1999. Phylogeny and taxonomy of the family Arthrodermataceae (dermatophytes) using sequence analysis of the ribosomal ITS region. Med Mycol 37:105–114. 10361266

[55] Heidemann S, Monod M, Gräser Y. 2010. Signature polymorphisms in the internal transcribed spacer region relevant for the differentiation of zoophilic and anthropophilic strains of Trichophyton interdigitale and other species of T. mentagrophytes sensu lato. Br J Dermatol 162:282–295. doi:10.1111/j.1365-2133.2009.09494.x 19886885

[56] Jabet A, Hamane S, Cremer G, Foulet F, Blaize M, Dellière S, Bonnal C, Imbert S, Brun S, Packeu A, Bretagne S, Fungi PRJ. 1103. MALDI-TOF mass spectrometry online identification of Trichophyton indotineae using the MSI-2 application. J Fungi (Basel) 8 10.3390/jof8101103 PMC9604624 36294668

[57] Nenoff P, Uhrlaß S, Verma SB, Panda S. 2022. Trichophyton mentagrophytes ITS genotype VIII and Trichophyton indotineae: a terminological maze, or is it? Indian J Dermatol Venereol Leprol 88:586–589. doi:10.25259/IJDVL_112_2022 35962516

[58] Caplan AS, Todd GC, Zhu Y, Sikora M, Akoh CC, Jakus J, Lipner SR, Graber KB, Acker KP, Morales AE, Rolón RMM, Westblade LF, Fonseca M, Cline A, Gold JAW, Lockhart SR, Smith DJ, Chiller T, Greendyke WG, Manjari SR, Banavali NK, Chaturvedi S. 2024. Clinical course, antifungal susceptibility, and genomic sequencing of Trichophyton indotineae. JAMA Dermatol 160:701–709. doi:10.1001/jamadermatol.2024.1126 38748419 PMC11097098

[59] Kumar P, Das S, Tigga R, Pandey R, Bhattacharya SN, Taneja B. 2021. Whole genome sequences of two Trichophyton indotineae clinical isolates from India emerging as threats during therapeutic treatment of dermatophytosis. 3 Biotech 11:402. doi:10.1007/s13205-021-02950-1 PMC8353060 34458064

[60] Teo JWP, Cheng JWS, Chew KL, Lin RTP. 2024. Whole genome characterization of Trichophyton indotineae isolated in Singapore. Med Mycol 62:myae012. doi:10.1093/mmy/myae012 38366631

[61] Yamada T, Yaguchi T, Maeda M, Alshahni MM, Salamin K, Guenova E, Feuermann M, Monod M. 2022. Gene amplification of CYP51B: a new mechanism of resistance to azole compounds in Trichophyton indotineae. Antimicrob Agents Chemother 66:e0005922. doi:10.1128/aac.00059-22 35546111 PMC9211412

[62] Kong X, Tang C, Singh A, Ahmed SA, Al-Hatmi AMS, Chowdhary A, Nenoff P, Gräser Y, Hainsworth S, Zhan P, Meis JF, Verweij PE, Liu W, de Hoog GS. 2021. Antifungal susceptibility and mutations in the squalene epoxidase gene in dermatophytes of the Trichophyton mentagrophytes species complex. Antimicrob Agents Chemother 65:e0005621. doi:10.1128/AAC.00056-21 33972254 PMC8284460

[63] Vincent YM, Musa J, Hasan MR, Leto D, Almohri H. 2023. Isolation of an emerging Trichophyton species in an Ontario community laboratory. Poster #214, Abstr 2023 AMMI Canada-CACMID Annual Conference; Toronto

[64] Spivack S, Gold JAW, Lockhart SR, Anand P, Quilter LAS, Smith DJ, Bowen B, Gould JM, Eltokhy A, Gamal A, Retuerto M, McCormick TS, Ghannoum MA. 2024. Potential sexual transmission of antifungal-resistant Trichophyton indotineae. Emerg Infect Dis 30:807–809. doi:10.3201/eid3004.240115 38437706 PMC10977831

[65] Verma SB, Panda S, Nenoff P, Singal A, Rudramuruthy SM, Uhrlass S, Das A, Bisherwal K, Shaw D, Vasani R. 2021. The unprecedented epidemic-like scenario of dermatophytosis in India: I. Epidemiology, risk factors and clinical features. IJDVL 87:154–175. doi:10.25259/IJDVL_301_20 33769736

[66] Martinez-Rossi NM, Peres NTA, Rossi A. 2017. Pathogenesis of dermatophytosis: sensing the host tissue. Mycopathologia 182:215–227. doi:10.1007/s11046-016-0057-9 27590362

[67] Khurana A, Sharath S, Sardana K, Chowdhary A. 2024. Clinico-mycological and therapeutic updates on cutaneous dermatophytic infections in the era of Trichophyton indotineae. J Am Acad Dermatol 91:315–323. doi:10.1016/j.jaad.2024.03.024 38574764

[68] Association AAoD. Recognizing Trichophyton Indotineae. Available from: https://www.aad.org/member/clinical-quality/clinical-care/emerging-diseases/dermatophytes/recognizing-trichophyton-indotineae. Retrieved 13 Dec 2024.

[69] Xu Z, Caplan AS. 2024. Extensive Tinea Corporis and Tinea Cruris from Trichophyton indotineae. N Engl J Med 391:1837–1837. doi:10.1056/NEJMicm2409010 39530420

[70] Crotti S, Cruciani D, Spina S, Piscioneri V, Natalini Y, Pezzotti G, Sabbatucci M, Papini M. 2023. A terbinafine sensitive Trichophyton indotineae strain in Italy: the first clinical case of tinea corporis and onychomycosis. J Fungi (Basel) 9:865. doi:10.3390/jof9090865 37754973 PMC10532841

[71] Jabet A, Monfort J-B, Hennequin C, Fekkar A, Piarroux R, Normand A-C. 2025. Trichophyton indotineae infection with toenail onychomycosis. Clin Exp Dermatol. doi:10.1093/ced/llaf089 39988977

[72] Pavlović MD, Marzouk S, Bećiri L. 2024. Widespread dermatophytosis in a healthy adolescent: the first report of multidrug-resistant Trichophyton indotineae infection in the UAE. Acta Dermatovenerol Alp Pannonica Adriat 33:53–55. 38347717

[73] Singh S, Chandra U, Anchan VN, Verma P, Tilak R. 2020. Limited effectiveness of four oral antifungal drugs (fluconazole, griseofulvin, itraconazole and terbinafine) in the current epidemic of altered dermatophytosis in India: results of a randomized pragmatic trial*. Br J Dermatol 183:840–846. doi:10.1111/bjd.19146 32538466

[74] Burmester A, Hipler UC, Hensche R, Elsner P, Wiegand C. 2019. Point mutations in the squalene epoxidase gene of Indian ITS genotype VIII T. mentagrophytes identified after DNA isolation from infected scales. Med Mycol Case Rep 26:23–24. doi:10.1016/j.mmcr.2019.09.001 31667055 PMC6812012

[75] Sacheli R, Egrek S, El Moussaoui K, Darfouf R, Adjetey AB, Hayette MP. 2024. Evaluation of currently available laboratory methods to detect terbinafine resistant dermatophytes including a gradient strip for terbinafine, EUCAST microdilution E.Def 11.0, a commercial real-time PCR assay, squalene epoxidase sequencing and whole genome sequencing. Mycoses 67:e70005. doi:10.1111/myc.70005 39658811

[76] Caplan AS, Zakhem GA, Pomeranz MK. 2023. Trichophyton mentagrophytes internal transcribed spacer genotype VIII. JAMA Dermatol 159:1130. doi:10.1001/jamadermatol.2023.2645 37418257

[77] Khurana A, Sardana K. 2018. Reinterpreting minimum inhibitory concentration (MIC) data of itraconazole versus terbinafine for dermatophytosis - time to look beyond the MIC data? Indian J Dermatol Venereol Leprol 84:61–62. doi:10.4103/ijdvl.IJDVL_715_17 29243676

[78] Khurana A, Agarwal A, Agrawal D, Panesar S, Ghadlinge M, Sardana K, Sethia K, Malhotra S, Chauhan A, Mehta N. 2022. Effect of different itraconazole dosing regimens on cure rates, treatment duration, safety, and relapse rates in adult patients with tinea corporis/cruris: a randomized clinical trial. JAMA Dermatol 158:1269–1278. doi:10.1001/jamadermatol.2022.3745 36103158 PMC9475442

[79] Gupta AK, Polla Ravi S, Wang T, Cooper EA, Lincoln SA, Foreman HC, Bakotic WL. 2023. Antifungal resistance, susceptibility testing and treatment of recalcitrant dermatophytosis caused by Trichophyton indotineae: a North American perspective on management. Am J Clin Dermatol 24:927–938. doi:10.1007/s40257-023-00811-6 37553539

[80] Siopi M, Efstathiou I, Theodoropoulos K, Pournaras S, Meletiadis J. 2021. Molecular epidemiology and antifungal susceptibility of Trichophyton isolates in Greece: emergence of terbinafine-resistant Trichophytonmentagrophytes type VIII locally and globally. J Fungi (Basel) 7:419. doi:10.3390/jof7060419 34072049 PMC8229535

[81] Sonego B, Corio A, Mazzoletti V, Zerbato V, Benini A, di Meo N, Zalaudek I, Stinco G, Errichetti E, Zelin E. 2024. Trichophyton indotineae, an emerging drug-resistant dermatophyte: a review of the treatment options. J Clin Med 13:3558. doi:10.3390/jcm13123558 38930086 PMC11204959

[82] Astvad KMT, Hare RK, Jørgensen KM, Saunte DML, Thomsen PK, Arendrup MC. 2022. Increasing terbinafine resistance in danish Trichophyton isolates 2019-2020. J Fungi (Basel) 8:150. doi:10.3390/jof8020150 35205904 PMC8879722

[83] Gupta AK, Venkataraman M, Hall DC, Cooper EA, Summerbell RC. 2023. The emergence of Trichophyton indotineae: implications for clinical practice. Int J Dermatol 62:857–861. doi:10.1111/ijd.16362 35867962

[84] Yamada T, Maeda M, Nagai H, Salamin K, Chang Y-T, Guenova E, Feuermann M, Monod M. 2023. Two different types of tandem sequences mediate the overexpression of TinCYP51B in azole-resistant Trichophyton indotineae. Antimicrob Agents Chemother 67:e0093323. doi:10.1128/aac.00933-23 37823662 PMC10648874

[85] Kovitwanichkanont T, Chong AH. 2019. Superficial fungal infections. Aust J Gen Pract 48:706–711. doi:10.31128/AJGP-05-19-4930 31569324

[86] Ely JW, Rosenfeld S, Seabury Stone M. 2014. Diagnosis and management of tinea infections. Am Fam Physician 90:702–710. 25403034 PMC12707599

[87] Anton A, Plinet M, Peyret T, Cazaudarré T, Pesant S, Rouquet Y, Tricoteaux MA, Bernier M, Bayette J, Fournier R, Marguerettaz M, Rolland P, Bayol T, Abbaoui N, Berry A, Iriart X, Cassaing S, Chauvin P, Bernard E, Fabre R, François JM. 2023. Rapid and accurate diagnosis of dermatophyte infections using the DendrisCHIP technology. Diagnostics (Basel) 13:3430. doi:10.3390/diagnostics13223430 37998565 PMC10670032

[88] Tsai T-F, Fan Y-C, Lu J-J, Chien C-C, Wang H-Y, Sun P-L. 2025. Identification of challenging dermatophyte species using matrix-assisted laser desorption/ionization time-of-flight mass spectrometry. J Fungi (Basel) 11:107. doi:10.3390/jof11020107 39997401 PMC11856511

[89] De Paepe R, Normand A-C, Uhrlaß S, Nenoff P, Piarroux R, Packeu A. 2024. Resistance profile, terbinafine resistance screening and MALDI-TOF MS identification of the emerging pathogen Trichophyton indotineae. Mycopathologia 189:29. doi:10.1007/s11046-024-00835-4 38483637 PMC10940462

[90] Normand A-C, Blaize M, Imbert S, Packeu A, Becker P, Fekkar A, Stubbe D, Piarroux R. 2021. Identification of molds with matrix-assisted laser desorption ionization-time of flight mass spectrometry: performance of the newly developed MSI-2 application in comparison with the bruker filamentous fungi database and MSI-1. J Clin Microbiol 59:e0129921. doi:10.1128/JCM.01299-21 34319807 PMC8451417

[91] Batvandi A, Pchelin IM, Kiasat N, Kharazi M, Mohammadi R, Zomorodian K, Rezaei-Matehkolaei A. 2023. Time and cost-efficient identification of Trichophyton indotineae. Mycoses 66:75–81. doi:10.1111/myc.13530 36114817

[92] Baron A, Hamane S, Gits-Muselli M, Legendre L, Benderdouche M, Mingui A, Ghelfenstein-Ferreira T, Alanio A, Dellière S. 2024. Dual quantitative PCR assays for the rapid detection of Trichophyton indotineae from clinical samples. Med Mycol Open Access 62. doi:10.1093/mmy/myae067 38977869

[93] Hwang JK, Gold JAW, Paller AS, Lipner SR. 2023. Low utilization of confirmatory testing for tinea capitis by pediatricians at an academic center in New York, United States, 2005–2021. Front Pediatr 11:1297339. doi:10.3389/fped.2023.1297339 38046680 PMC10690948

[94] Singh A, Singh P, Dingemans G, Meis JF, Chowdhary A. 2021. Evaluation of DermaGenius^®^ resistance real-time polymerase chain reaction for rapid detection of terbinafine-resistant Trichophyton species. Mycoses 64:721–726. doi:10.1111/myc.13271 33760310

[95] Yamada T, Maeda M, Alshahni MM, Tanaka R, Yaguchi T, Bontems O, Salamin K, Fratti M, Monod M. 2017. Terbinafine resistance of Trichophyton clinical isolates caused by specific point mutations in the squalene epoxidase gene. Antimicrob Agents Chemother 61:e00115-17. doi:10.1128/AAC.00115-17 PMC5487658 28416557

[96] Arendrup M.C, Jørgensen KM, Guinea J, Lagrou K, Chryssanthou E, Hayette MP, Barchiesi F, Lass-Flörl C, Hamal P, Dannaoui E, Chowdhary A, Hare RK, Meletiadis J. 2022. Comment on: multicentre validation of a EUCAST method for the antifungal susceptibility testing of microconidia-forming dermatophytes. J Antimicrob Chemother 77:1212–1213. doi:10.1093/jac/dkac004 35075481

[97] Arendrup M.C, Jørgensen KM, Guinea J, Lagrou K, Chryssanthou E, Hayette MP, Barchiesi F, Lass-Flörl C, Hamal P, Dannaoui E, Chowdhary A, Meletiadis J. 2020. Multicentre validation of a EUCAST method for the antifungal susceptibility testing of microconidia-forming dermatophytes. J Antimicrob Chemother 75:1807–1819. doi:10.1093/jac/dkaa111 32303059

[98] Arendrup MC, Kahlmeter G, Guinea J, Meletiadis J, Subcommittee on Antifungal Susceptibility Testing (AFST) of the ESCMID European Committee for Antimicrobial Susceptibility Testing (EUCAST). 2021. How to: perform antifungal susceptibility testing of microconidia-forming dermatophytes following the new reference EUCAST method E.Def 11.0, exemplified by Trichophyton. Clin Microbiol Infect 27:55–60. doi:10.1016/j.cmi.2020.08.042 32916260

[99] Schirmer H, Henriques C, Simões H, Veríssimo C, Sabino R. 2025. Prevalence of T. rubrum and T. interdigitale exhibiting high MICs to terbinafine in clinical samples analyzed in the portuguese mycology reference laboratory. Pathogens 14:115. doi:10.3390/pathogens14020115 40005492 PMC11858771

[100] Bidaud AL, Moreno-Sabater A, Normand AC, Cremer G, Foulet F, Brun S, Ayachi A, Imbert S, Chowdhary A, Dannaoui E. 2023. Evaluation of gradient concentration strips for detection of terbinafine resistance in Trichophyton spp. Antimicrob Agents Chemother 67:e0171622. doi:10.1128/aac.01716-22 37162356 PMC10269145

[101] Sahni K, Singh S, Dogra S. 2018. Newer topical treatments in skin and nail dermatophyte infections. Indian Dermatol Online J 9:149. doi:10.4103/idoj.IDOJ_281_17 29854633 PMC5956860

[102] Goldstein AG. n.d. Dermatophyte(tinea) infections. In UpToDate. Wolters Kluwer.

[103] Kassem MAA, Esmat S, Bendas ER, El-Komy MHM. 2006. Efficacy of topical griseofulvin in treatment of tinea corporis. Mycoses 49:232–235. doi:10.1111/j.1439-0507.2006.01221.x 16681816

[104] Pires CAA, Cruz NFS da, Lobato AM, Sousa PO de, Carneiro FRO, Mendes AMD. 2014. Clinical, epidemiological, and therapeutic profile of dermatophytosis. An Bras Dermatol 89:259–264. doi:10.1590/abd1806-4841.20142569 24770502 PMC4008056

[105] Moreno-Sabater A, Normand AC, Bidaud AL, Cremer G, Foulet F, Brun S, Bonnal C, Aït-Ammar N, Jabet A, Ayachi A, Piarroux R, Botterel F, Houzé S, Desoubeaux G, Hennequin C, Dannaoui E. 2022. Terbinafine resistance in dermatophytes: a French multicenter prospective study. J Fungi (Basel) 8:220. doi:10.3390/jof8030220 35330222 PMC8948947

[106] Khurana A, Masih A, Chowdhary A, Sardana K, Borker S, Gupta A, Gautam RK, Sharma PK, Jain D. 2018. Correlation of in vitro susceptibility based on MICs and squalene epoxidase mutations with clinical response to terbinafine in patients with tinea corporis/cruris. Antimicrob Agents Chemother 62:e01038-18. doi:10.1128/AAC.01038-18 30275090 PMC6256768

[107] Gawaz A, Nenoff P, Uhrlaß S, Schaller M. 2021. Treatment of a terbinafine-resistant trichophyton mentagrophytes type VIII. Hautarzt 72:900–904. doi:10.1007/s00105-021-04857-7 34241651

[108] Cañete-Gibas CF, Mele J, Patterson HP, Sanders CJ, Ferrer D, Garcia V, Fan H, David M, Wiederhold NP. 2023. Terbinafine-resistant dermatophytes and the presence of Trichophyton indotineae in North America. J Clin Microbiol 61:e0056223. doi:10.1128/jcm.00562-23 37432126 PMC10446870

[109] Caplan AS, Chaturvedi S, Todd G, Sikora M, Ugwu-Dike PO, Pahalyants V, Taiwo D, Gold JAW. 2025. Response to "Clinico-mycological and therapeutic updates on cutaneous dermatophytic infections in the era of Trichophyton indotineae"; Focus on griseofulvin. J Am Acad Dermatol 92:e11–e12. doi:10.1016/j.jaad.2024.06.109 39245365 PMC11663085

[110] Gueneau R, Joannard B, Haddad N, Alby F, Jullien V, Schlatter J, Cotteret C, Bougnoux ME, Lanternier F, Laroche L, Delliere S, Cisternino S, Lortholary O. 2022. Extensive dermatophytosis caused by terbinafine-resistant Trichophyton indotineae, successfully treated with topical voriconazole. Int J Antimicrob Agents 60:106677. doi:10.1016/j.ijantimicag.2022.106677 36184016

[111] Galili E, Lubitz I, Shemer A, Astman N, Pevzner K, Gazit Z, Segal O, Lyakhovitsky A, Halevi S, Baum S, Barzilai A, Amit S. 2024. First reported cases of terbinafine-resistant trichophyton indotineae isolates in israel: epidemiology, clinical characteristics and response to treatment. Mycoses 67:e13812. doi:10.1111/myc.13812 39547945

